# Utility of VS38c in the diagnostic and prognostic assessment of osteosarcoma and other bone tumours/tumour-like lesions

**DOI:** 10.1186/s13569-017-0083-5

**Published:** 2017-09-18

**Authors:** E. S. Hookway, Z. Orosz, Y. Uchihara, A. Grigoriadis, A. B. Hassan, U. Oppermann, N. A. Athanasou

**Affiliations:** 1 Nuffield Department of Orthopaedics, Rheumatology and Musculoskeletal and Sciences, University of Oxford, Nuffield Orthopaedic Centre, Oxford, OX3 7HE UK; 2 Department of Craniofacial Development and Stem Cell Biology, Guy’s Hospital, King’s College, London, UK

**Keywords:** Osteosarcoma, VS38c, Rough endoplasmic reticulum, Bone tumour, Therapy

## Abstract

**Background:**

VS38c is a monoclonal antibody that recognises a rough endoplasmic reticulum (rER) intracellular antigen termed cytoskeleton-linking membrane protein 63. rER is typically found in viable tumour cells and is abundant in osteosarcoma cells. The aim of this study was to determine the diagnostic and prognostic utility of VS38c in the histological assessment of osteosarcoma and other bone tumours/tumour-like leisons.

**Methods:**

Immunohistochemical staining with VS38c was carried out on formalin-fixed specimens of osteosarcoma (pre/post-chemotherapy) and a wide range of benign and malignant bone lesions. In addition, VS38c staining of cultures of MG63 and Sa0S2 osteosarcoma cell cultures. (±cisplatin and actinomycin D-treatment) was analysed.

**Results:**

VS38c strongly stained tumour cells in all low-grade and high-grade osteosarcomas and in undifferentiated sarcomas and high-grade chondrosarcomas. There was little or no VS38c staining of low-grade chondrosarcomas or chordomas and variable staining of Ewing sarcomas. Osteoblasts in benign bone-forming tumours and mononuclear stromal cells in chondroblastomas, giant cell tumours and non-ossifying fibromas strongly stained for VS38c. VS38c staining was absent in cisplatin and actinomycin D treated Sa0S2 and MG63 cells. In specimens of osteosarcoma post-neoadjuvant therapy, VS38c staining was absent in most morphologically necrotic areas of tumor although some cells with pyknotic nuclei stained for VS38c in these areas. Most tumour cells exhibiting atypical nuclear forms were not stained by VS38c.

**Conclusions:**

Our findings show that VS38c is a sensitive but not specific diagnostic marker of osteosarcoma. Staining with VS38c identifies viable osteosarcoma cells, a feature which may be useful in the assessment of percentage tumour necrosis post-neoadjuvant chemotherapy.

## Background

VS38c is a monoclonal antibody that recognises an intracellular antigen identified as the rough endoplasmic reticulum (rER) cytoskeleton-linking membrane protein P63 (CLIMP 63) which has also been termed P63 and cytoskeletal-associated protein 4 [1]. CLIMP-63 is a stable, abundant rER protein that plays a role in protein transport. The VS38c monoclonal antibody strongly stains immunoglobulin-producing plasma cells and facilitates the immunohistochemical diagnosis of myeloma [2, 3]. VS38c staining of tumour cells in neuroendocrine carcinoma, melanoma and osteosarcoma has also been reported [4–7]. A common feature of all tumour cells that stain with VS38c is that they ultrastructurally contain numerous cytoplasmic ribosomes and abundant rER.

Regular protein production is a functional characteristic of viable cells, and loss or inactivation of ribosomes and rER has been noted in necrotic tumour cells [8, 9]. Several types of cell death involve changes in ER [10, 11], and ER stress has been shown to play a role in the necrosis of osteosarcoma cells [12–14]. Osteosarcomas are usually treated pre-operatively by a course of neoadjuvant chemotherapy with assessment of the response evaluated by determining histologically the extent of necrosis in the resected tumour specimen [15–19]; this percentage measure of tumour necrosis provides important prognostic information and gives a guide to as to whether adjuvant chemotherapy is required. Osteosarcoma cells can exhibit nuclear abnormalities post-chemotherapy and it is not certain whether such cells are viable or apoptotic in this context [20–26]. There are no formal morphological criteria that can be used to determine whether an osteosarcoma cell is viable or not and it can be difficult to assess accurately the extent of necrosis in an osteosarcoma specimen.

In this study, we have sought to determine the diagnostic and prognostic utility of VS38c in the assessment of osteosarcoma and other primary bone tumours. We have examined VS38c staining in a wide range of benign and malignant bone tumours, including osteosarcoma subtypes, in order to assess the sensitivity and specificity of this marker for the diagnosis of osteosarcoma. In addition, as ER changes have been noted in necrotic tumour cells, and active protein production is a feature of cell viability, we have examined VS38c staining in osteosarcomas that have received neoadjuvant chemotherapy in order to determine if expression of this marker can be used to identify viable tumour cells and to facilitate the evaluation of percentage tumour necrosis in osteosarcoma specimens.

## Methods

### Specimens analysed

Paraffin-embedded tissue from 178 specimens of bone tumours as well as specimens of normal compact and cancellous bone, femoral articular surface and growth plate, were retrieved from the files of the Nuffield Orthopedic Centre Histopathology Department, Oxford (Table 1). 61 cases of high-grade osteosarcoma were analysed including 25 cases that had histology pre/post-neoadjuvant chemotherapy. Criteria for the histological diagnosis of the bone tumours were those of the 2013 WHO Classification of Tumours of Soft Tissue and Bone [27]. Samples were derived from biopsies and resection specimens fixed in 10% buffered formalin and decalcified in 5% nitric acid. Histology was assessed using a Nikon Eclipse 80i microscope.

**Table 1:** VS38c staining of benign and malignant bone tumours/tumour-like lesions

Osteoid osteoma	(4)
Osteoblastoma	(4)
Osteosarcoma—high-grade	
Osteoblastic	(46)
Chondroblastic	(6)
Telangiectatic	(6)
Giant-cell rich	(1)
Small cell	(2)
Osteosarcoma—low grade	
Intramedullary	(2)
Parosteal	(4)
Fibrous dysplasia^a^	(8)
Osteochondroma^a^	(3)
Enchondroma^a^	(6)
Chondroblastoma	(4)
Chondromyxoid fibroma^a^	(3)
Atypical cartilaginous tumour/Chondrosarcoma, Grade I	(12)
Chondrosarcoma, Grade II/III	(8)
Ewing sarcoma^a^	(16)
Giant cell tumour of bone	(12)
Aneurysmal bone cyst	(4)
Simple bone cyst	(2)
Osteofibrous dysplasia	(4)
Adamantinoma	(3)
Chordoma^a^	(6)
Undifferentiated sarcoma	(4)
Langerhans cell histiocytosis^a^	(4)
Fracture	(2)
Myositis ossificans	(2)

(), number of cases

^a^Tumours where fewer than 10% lesional stained cells with VS38c

### Immunohistochemistry

5 μm sections of formalin-fixed paraffin-embedded tissue were cut and then stained by an indirect immunoperoxidase technique using chemMate Envision (Dako, UK). After blocking endogenous peroxidase, sections were incubated with the monoclonal antibody VS38C (Dako, UK) with antigen unmasking pretreatment. Antigens were detected by incubation with labelled polymer and diaminobenzidine. Sections of myeloma were employed as a positive control. Negative controls consisted of sections which were incubated with normal mouse IgG1 (10 or 20 μg/mL, Sigma-Aldrich, Dorset, UK) substituted for the monoclonal antibody. The VS38c staining was scored as strong or weak relative to the positive control. Where the majority of lesional cells were not stained with VS38c, an assessment of the percentage of VS38-positive cells relative to the total number of (lesional) cells was noted.

### Expression of VS38c in MG63 and Sa0S2 cells

MG63 and SAOS2 human osteosarcoma cells were cultured in Iscove’s Modified Dulbecco’s Medium (Sigma-Aldrich, Dorset, UK) supplemented with 10% fetal calf serum [28]. To determine expression of VSC38c, cells were plated at either low (10^5^ cells) or high (10^6^ cells) density in a 6 well plate and allowed to adhere overnight. Cells were then treated for 24 h in the presence of either cisplatin (1 µg/mL), actinomycin D (10 µg/mL) or vehicle control. Cultures were fixed in formalin and stained as above by immunohistochemistry with the antibody VS38c.

## Results

### VS38c staining of normal bone and cartilage

**Fig. 1:**
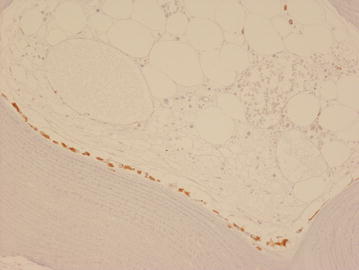
Immunohistochemistry with monoclonal antibody VS38c showing strong staining of osteoblasts in remodelling cancellous bone. (original magnification ×200)

Strong cytoplasmic staining of osteoblasts with VS38c was noted in compact and cancellous woven and lamellar bone in areas of bone remodelling (Fig. 1). A few osteocytes in woven bone and a few marrow stromal cells and flattened bone lining cells were also weakly VS38c positive. Osteoclasts and fat cells in bone, fibroblasts in periosteal dense connective tissue, and endothelial cells/smooth muscle cells in blood vessels were negative for VS38c. Chondrocytes in hyaline articular cartilage and in the reserve and proliferating zones of growth plate cartilage were unstained; hypertrophic and mineralizing cartilage cells in the growth plate stained with VS38c.

### VS38c staining of osteosarcoma and other bone-forming tumours and tumour-like lesions

**Fig. 2:**
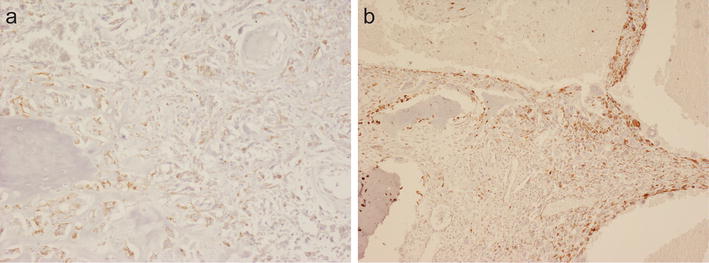
Immunohistochemistry with monoclonal antibody VS38c showing strong staining of osteoid-forming tumour cells in: (**a**) high-grade osteoblastic osteosarcoma and (**b**) telangiectatic osteosarcoma. (original magnification: **a** ×200; **b** ×100)

Tumour cells in all high-grade osteoblastic osteosarcomas (Fig. 2a), as well as small cell and telangiectatic (Fig. 2b) sub-types strongly stained for VS38c; tumour cells in chondroblastic and fibroblastic areas of high-grade osteosarcoma were also strongly stained by VS38c. VS38c also strongly stained osteogenic tumour cells in intramedullary low-grade osteosarcoma and parosteal osteosarcoma. In other primary bone-forming tumours, osteoid osteoma and osteoblastoma, there was also strong VS38c staining of osteoblasts lining the surface of woven bone; some newly formed woven bone trabeculae in these lesions also showed VS38c staining of osteocytes. In four cases of fibrous dysplasia, there was strong VS38c staining of mononuclear stromal cells in the intertrabecular fibrous tissue, the remaining cases showing little (<10%) or no staining. In osteofibrous dysplasia, VS38c strongly stained plump osteoblasts rimming woven bone and stromal cells in the fibrous stroma. Osteoblasts in reactive woven bone in fracture callus and myositis ossificans were also strongly VS38c positive.

### VS38c staining of other primary bone tumours

**Fig. 3:**
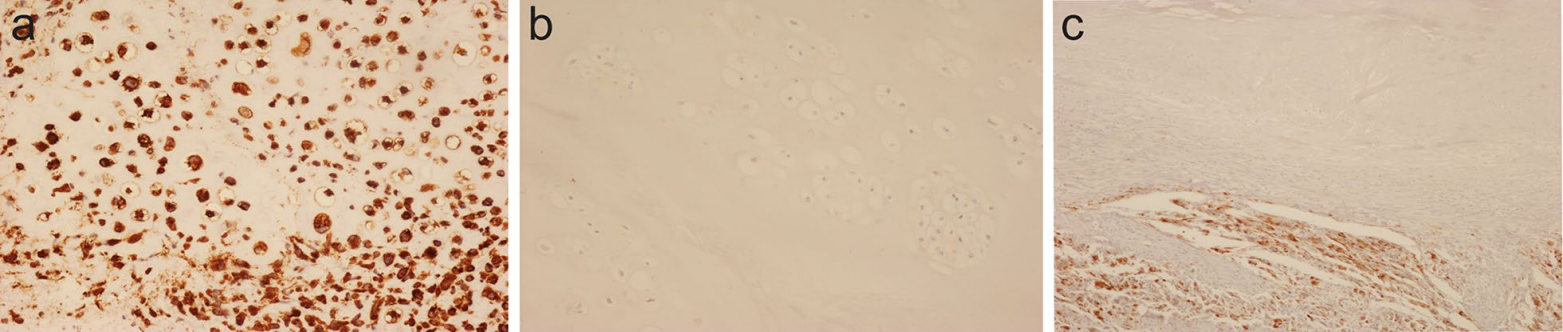
Immunohistochemistry with monoclonal antibody VS38c showing: **a** strong staining of cartilage tumour cells in high-grade chondrosarcoma; **b** absence of staining of cartilage tumour cells in low-grade chondrosarcoma; **c** in dedifferentiated chondrosarcoma absence of staining in the low-grade chondrosarcoma component and strong staining in the dedifferentiated pleomorphic sarcoma component. (original magnification: **a** ×400; **b** ×400; **c** ×100)

No staining or weak staining of a few (<10%) cartilage cells was noted with VS38c in benign cartilage tumours including enchondroma, osteochondroma and chondromyxoid fibroma; some cellular enchondromas of the hand, however, showed more prominent staining of cartilage cells. In contrast, VS38c strongly stained chondroblasts in chondroblastoma. Atypical cartilaginous tumour/chondrosarcoma grade 1 showed a similar pattern of VS38c staining to that seen in most enchondromas, but malignant cells in high-grade chondrosarcomas strongly and diffusely stained with VS38c (Fig. 3a, b). In dedifferentiated chondrosarcoma, the low-grade chondrosarcoma component showed little staining with VS38c whereas the high-grade sarcoma component was strongly stained (Fig. 3c).

**Fig. 4:**
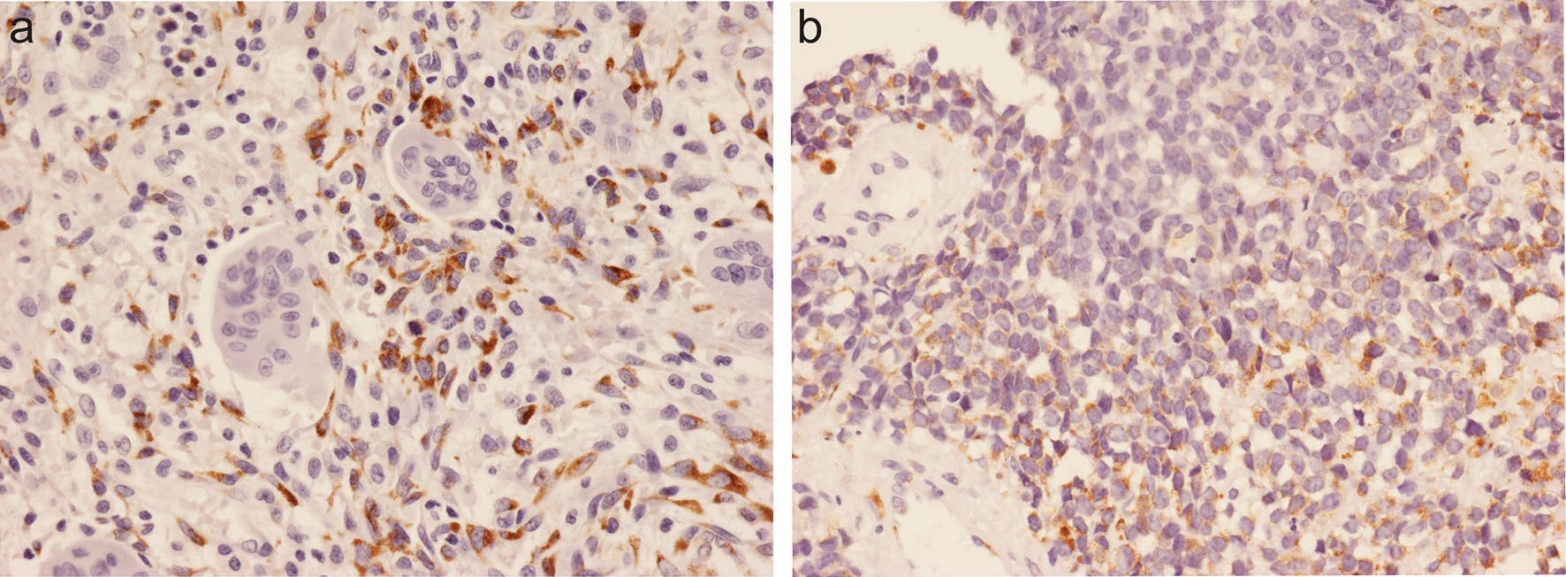
Immunohistochemistry with monoclonal antibody VS38c showing: **a** staining of mononuclear cells but not giant cells in giant cell tumour of bone; and **b** staining of tumour cells in Ewing sarcoma. (original magnification: **a** ×400; **b** ×400)

In giant cell tumour of bone, mononuclear cells strongly stained with VS38c but osteoclast-like giant cells were negative (Fig. 4a). A similar pattern of staining was noted in non-ossifying fibroma and aneurysmal bone cyst where reactive woven bone and scattered fibroblastic cells in the cyst wall were also stained by VS38c.

All tumor cells in primary high-grade undifferentiated spindle cell/pleomorphic sarcoma of bone stained strongly with VS38c. Ten of 16 cases of Ewing sarcoma were also strongly and diffusely stained with VS38c, (Fig. 4b), but six cases showed staining of a few (<10%) cells. Most chordomas were VS38c-negative but two cases showed weak staining of a few (<10%) cells. VS38c strongly stained adamantinomas in both epithelial and mesenchymal components.

### VS38c staining of osteosarcomas receiving neoadjuvant chemotherapy

**Fig. 5:**
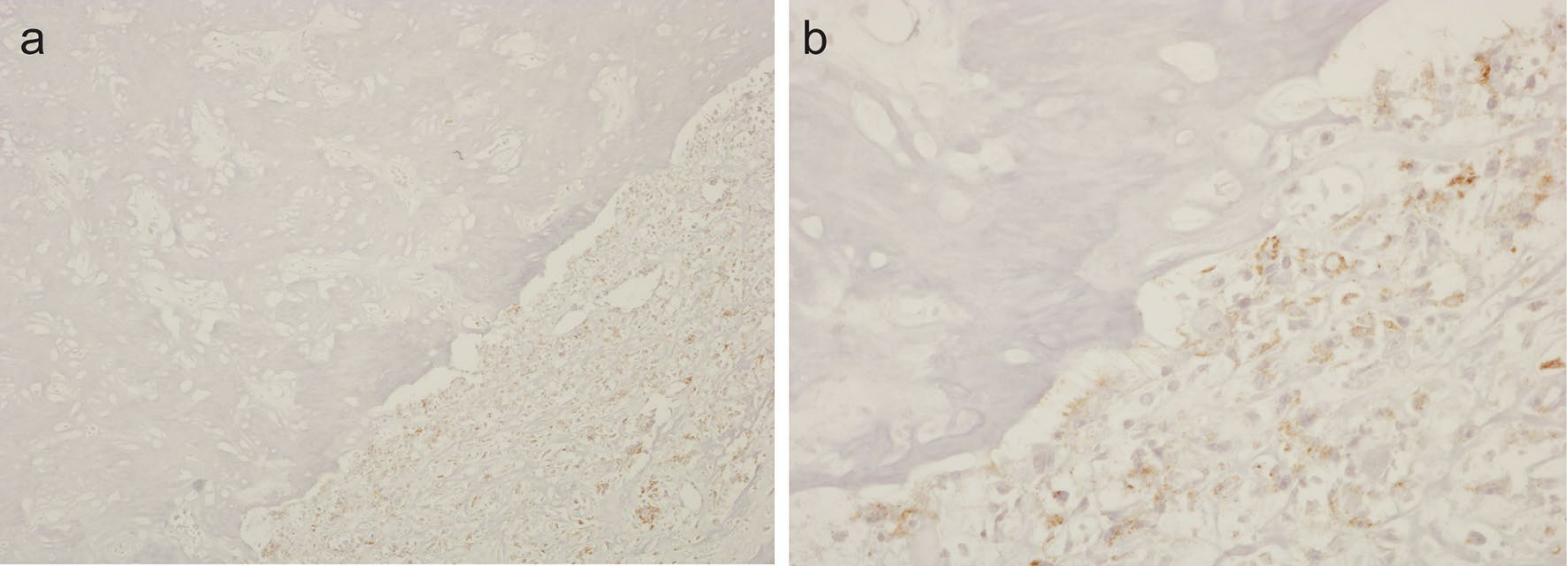
Immunohistochemistry with monoclonal antibody VS38c of an osteosarcoma that had received neoadjuvant therapy showing: **a** low and **b** high-power views of VS38c staining of residual viable tumour (right) and absence of staining in necrotic areas of tumour (left) (original magnification: **a** ×100; **b** ×400)

**Fig. 6:**
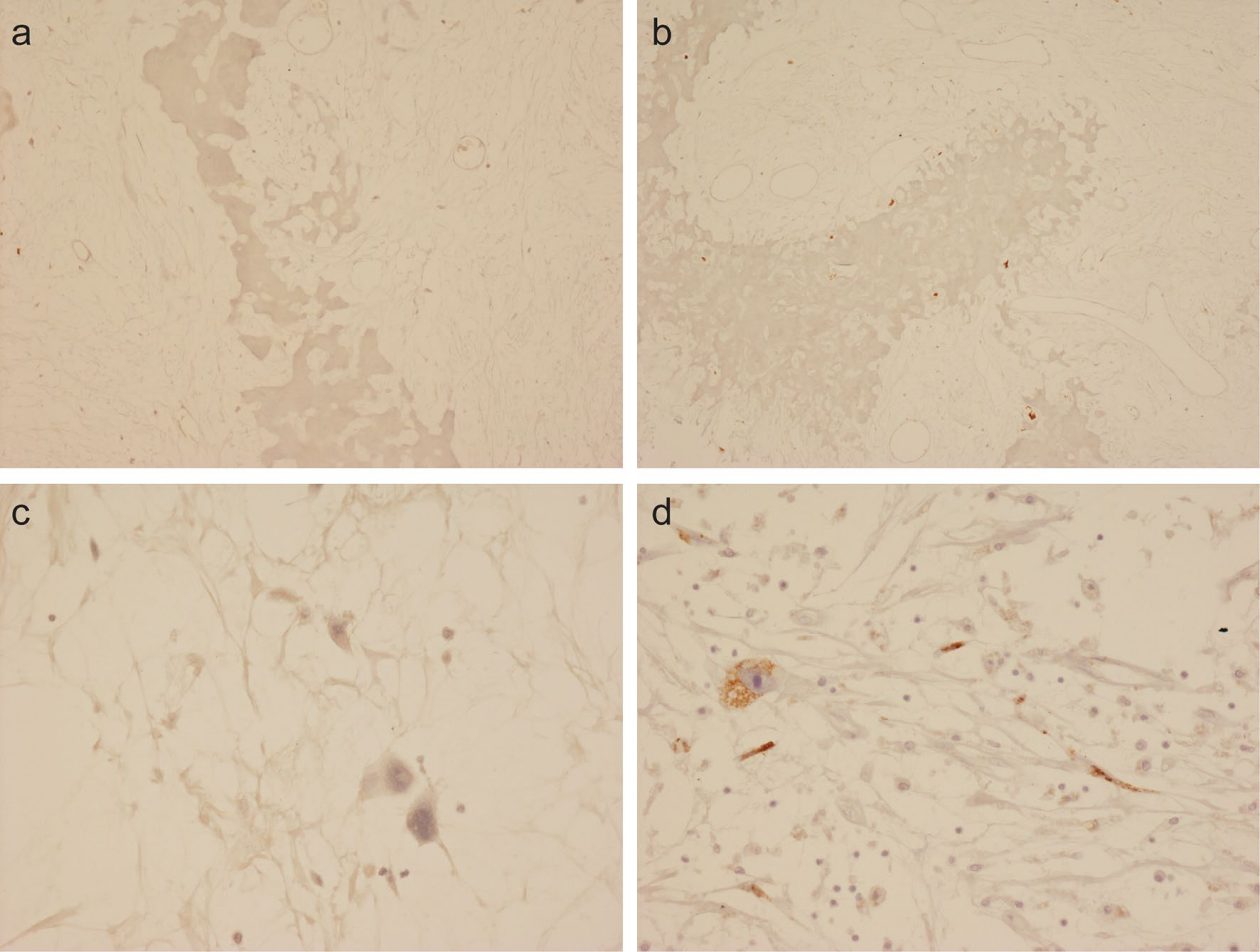
Immunohistochemistry with monoclonal antibody VS38c of an osteosarcoma that had received neoadjuvant therapy showing in necrotic areas of the tumour: **a** absence and **b** variable VS38c staining of cells with pyknotic nuclei and **c** absence and **d** variable staining with VS38c of atypical cells with vacuolated cytoplasm (original magnification: **a** ×200; **b** ×200; **c** ×400; **d** ×400)

Strong VS38c staining of osteosarcoma cells was noted in all pre-treatment biopsies of the 25 cases of osteosarcoma that had received pre-operative neoadjuvant therapy. VS38c strongly stained areas containing residual viable tumour in resection specimens of these treated osteosarcomas (Fig. 5). Absence of VS38c staining was noted in areas of tumour necrosis where there was only amorphous cellular debris or ghost cells i.e. where nucleated tumour cells could not be identified morphologically (Figs. 5, 6a). In morphologically necrotic areas of tumour, a variable number of cells with pyknotic nuclei stained with VS38c (Fig. 6b). In some tumours, there were scattered atypical cells with vacuolated cytoplasm and bizarre homogenization of nuclear chromatin; most of these cells were negative for VS38c (Fig. 6c) but a few were noted to be VS38c positive (Fig. 6d).

### VS38c staining of cultured osteosarcoma cells treated by cisplatin and actinomycin D

**Fig. 7:**
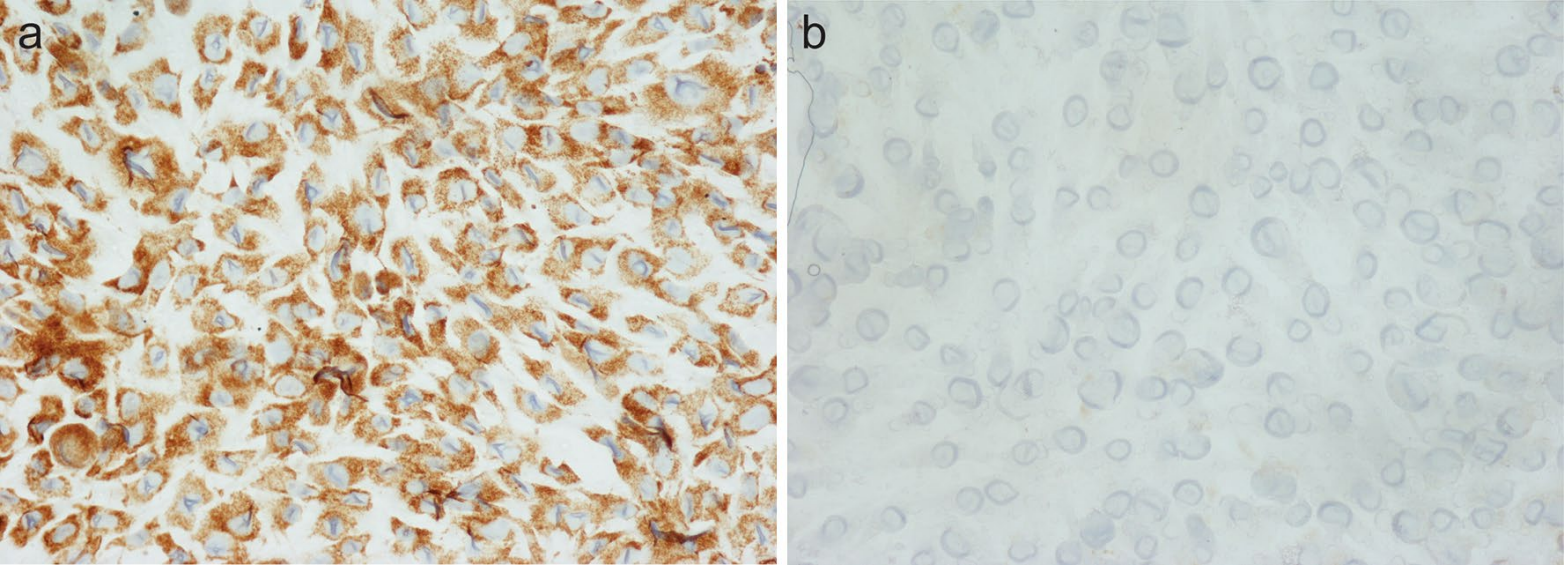
Immunohistochemistry with monoclonal antibody VS38c showing (**a**) staining of cultured SAOS2 osteosarcoma cells and (**b**) SAOS2 cells treated with cisplatin. (original magnification: **a** ×400; **b** ×400)

Control cultures of MG63 and Sa0S2 osteosarcoma cells contained viable tumour cells that strongly expressed VS38c (Fig. 7a). In parallel cultures to which cisplatin and actinomycin D had been added, there was necrosis of tumour cells which did not stain for VS38c (Fig. 7b).

## Discussion

Osteosarcoma is the most common primary malignant tumour of bone. Histological diagnosis of osteosarcoma is based on the identification of cytologically malignant tumour cells that form osteoid/bone. These matrix protein-producing malignant cells ultrastructurally contain numerous ribosomes and rER. In this study, we have shown that tumour cells in all high-grade and low-grade osteosarcomas are stained by the monoclonal antibody VS38c which recognises the CLIMP-63 protein on rER. Although absence of VS38c expression effectively excludes the diagnosis of osteosarcoma, the diagnostic specificity of this antibody is limited because, as noted previously [5], VS38c also stains other bone tumours. Absence of staining with VS38c was noted in cultures of actinomycin D/cisplatin-treated osteosarcoma cells and in necrotic areas of (post-neoadjuvant therapy) osteosarcoma specimens, a finding which may be useful in evaluating percentage tumour necrosis in osteosarcoma.

In this study we characterised the pattern of VS38c staining in primary benign and malignant bone tumours. In addition to osteosarcoma and other benign bone-forming tumours, VS38c strongly stained other high-grade primary malignant bone tumours, including undifferentiated spindle-cell/pleomorphic sarcoma and Grade II/III chondrosarcoma. VS38c staining was also noted in most Ewing sarcomas, although the number of stained cells was highly variable. Atypical cartilaginous tumour/chondrosarcoma grade 1 and chordoma were either negative or showed little staining with VS38c. In a number of giant-cell rich lesions of bone, including giant-cell tumour, non-ossifying fibroma, chondroblastoma and aneurysmal bone cyst, VS38c strongly stained mononuclear cells but not osteoclastic giant cells. VS38c staining of such a wide range of bone tumours clearly limits its diagnostic specificity but, given its strong and consistent staining of osteosarcoma, it may be useful as a highly sensitive if not specific positive marker of this tumour.

Although a number of factors, (e.g. tumour site, size and histological subtype) influence disease survival in osteosarcoma, the extent of chemotherapy-induced necrosis has been shown to correlate most strongly with prognosis. Multi-drug neoadjuvant chemotherapy for high-grade osteosarcoma is now routinely employed and systematic pathological evaluation of the resection specimen of a treated osteosarcoma has been shown to yield important prognostic information about the chemotherapeutic response and to provide a guide as to whether adjuvant chemotherapy is required [16–19]. In cases where chemotherapy-induced necrosis is 90% or more, the 5 year disease free survival is more than 80% [27, 29–36]. Loss of ribosomes and rER is seen in necrotic tumour cells [10, 11]. We noted that, in osteosarcoma specimens that had received neoadjuvant therapy, VS38c staining was strongly positive in areas of residual viable tumour but largely absent in morphologically necrotic areas of tumour; this pattern of staining was reflected in osteosarcoma cell cultures, where VS38c strongly stained viable but not necrotic tumour cells.

Qualitative assessment of the percentage area of tumour necrosis in pathological specimens of osteosarcoma is believed to correlate well with quantitative morphometric analysis [15–19]. However, there are no studies, to our knowledge, which have proved that morphological assessment of tumour necrosis in osteosarcoma is accurate. Most histopathologists rely on the identification of cytoplasmic and nuclear alterations indicative of cell death, such as pyknosis, karyorrhexis and karyolysis, to determine tumour necrosis in chemotherapy-treated osteosarcomas. Necrotic areas of tumour often contain ghost cells which show a variable degree of loss of nuclear and cytoplasmic detail. However, irreversible cell death or necrosis is difficult to distinguish morphologically from reversible degenerative change or apoptosis affecting individual cells or small groups of cells. Preoperative neo-adjuvant chemotherapy of osteosarcomas not only results in necrosis, but also changes in the tumour stroma which maybe partly calcified, contain hypermineralised bone or areas of reparative fibrosis, neovascularisation and inflammation [22–24, 26]. A major challenge to the surgical pathologist is the interpretation of atypical cells containing vacuolated cytoplasm and hyperchromatic nuclei with smudged or clumped chromatin found in some post-treatment osteosarcoma specimens. These atypical cells are often found in areas where there is not only necrosis but also fibrosis and it can be difficult to determine morphologically whether such cells are apoptotic/necrotic or viable. We found that most of these cells were VS38c negative, indicating that they did not contain rER. However, a few of these cells were VS38c positive. Cells with pyknotic nuclei are not uncommonly seen in areas of tumour necrosis in post-chemotherapy osteosarcoma specimens. Pyknosis is a nuclear feature seen in both apoptotic and necrotic cells and we noted that some tumour cells with pyknotic nuclei were VS38c positive; this indicates that such cells contain rER and that they are thus potentially capable of protein production, a feature characteristic of viable tumour cells.

In conclusion, our findings indicate that VS38c is a highly sensitive but not specific diagnostic marker for osteosarcoma. VS38c staining was seen in other primary benign and malignant bone tumours but it was not prominent in some low-grade tumours such as atypical cartilaginous tumour. VS38c staining outlined areas of residual viable tumour in post-chemotherapy osteosarcoma specimens and aided assessment of percentage tumor necrosis. However, although there was little or no VS38c staining in most areas of tumour necrosis in osteosarcoma specimens, there was variable staining of atypical cells with clumped chromatin and vacuolated cytoplasm and cells with pyknotic nuclei. The status of these VS38c positive cells in these necrotic areas is uncertain and more studies are needed to determine whether the morphological assessment of percentage tumour necrosis truly reflects the extent of viable and necrotic tumour in osteosarcoma specimens.
